# Retraction: MicroRNA-363 inhibits ovarian cancer progression by inhibiting NOB1

**DOI:** 10.18632/oncotarget.28446

**Published:** 2023-06-12

**Authors:** Yang Lin, Tianmin Xu, Shunqing Zhou, Manhua Cui

**Affiliations:** ^1^Department of Obstetrics and Gynecology, The Second Hospital, Jilin University, Nanguan District, Changchun 130041, China

**This article has been retracted:** A number of figure images in this paper were previously published in earlier papers. Figure 2E contains images from Figure 6C of another paper [1]. Figure 5D also contains images from two other published papers which have since been retracted [2, 3]. Figure 2C also contains images from Figure 2C of another published paper [4]. Images in Figure 3B and 5F are sourced from another published paper [5]. Figure 6B also contains images from another published paper [6]. Figure 5B has an image from Figure 4D in another published paper [7]. In addition, the authors are unable to reproduce the results represented in the paper. As a result, all authors have agreed to this retraction.

Original article: Oncotarget. 2017; 8:101649–101658. 101649-101658. https://doi.org/10.18632/oncotarget.21417
